# Status of Notifiable Infectious Diseases Reporting in the Seoul Metropolitan Area, Republic of Korea, 2025

**DOI:** 10.56786/PHWR.2026.19.43.4

**Published:** 2026-11-12

**Authors:** Sungnam Kim, Jihye Choo, Hyunjung Kim, Mijeong Ko, Jonghee Kim

**Affiliations:** Division of Infectious Disease Control and Response, Capital Regional Center for Disease Control and Prevention, Korea Disease Control and Prevention Agency Seoul Korea

**Keywords:** Infectious diseases, Statistics, Public health, Capital Regional Center for Disease Control and Prevention

## Abstract

**Objectives:**

The Capital Regional Center for Disease Control and Prevention has jurisdiction over four cities and provinces (Seoul Metropolitan City, Incheon Metropolitan City, Gyeonggi province, and Gangwon State), which account for 53.9% of total population of the Republic of Korea. To analyze and summarize the prevalence and high incidence of notifiable infectious diseases reported in the Seoul Metropolitan Area in 2025. This study analyzes surveillance data to identify trends in infectious disease outbreaks in the Seoul Metropolitan Area and to inform the Capital Regional Center for Disease Control and Prevention's future policies on infectious disease prevention, management, preparedness, and response.

**Methods:**

Data on reported cases of notifiable infectious diseases were collected through the Infectious Disease Information System. Based on this, we analyzed 66 infectious diseases subject to mandatory surveillance.

**Results:**

In 2025, the Seoul Metropolitan Area reported 74,895 cases of notifiable infectious diseases subject to mandatory reporting, an 18.9% decrease from 2024. Carbapenem-resistant *Enterobacterales* (CRE) infection, varicella, and tuberculosis were the most common, while Mpox, malaria, and Lyme disease were high-proportion infectious diseases in the Seoul Metropolitan Area relative to the nationwide total.

**Conclusions:**

Infectious disease characteristics in the Seoul Metropolitan Area included a continuous increase in CRE infection, which is Healthcare-Associated Infections; a concentration of syphilis cases, a sexually transmitted infection; and a high proportion of imported infectious diseases. Furthermore, the Seoul Metropolitan Area accounts for approximately 90% of all malaria cases nationwide, with most cases occurring in the region. This study helps to understand infectious disease trends in the Area and develop policies for prevention, control, preparedness, and response. The center will strengthen its cooperative system with four provinces. By implementing field-based, immediate responses, it will help prevent infectious disease introduction and reduce further spread.

**Key messages**

**① What is known previously?**

The Capital Regional Center for Disease Control and Prevention oversees four provinces, including Seoul, Incheon, Gyeonggi, and Gangwon. It has established a surveillance system for the national notifiable infectious diseases and responded immediately.

**② What new information is presented?**

In 2025, the Seoul Metropolitan Area reported 74,895 cases of nationally notifiable infectious diseases subject to mandatory reporting, an 18.9% decrease from 2024. The characteristics of infectious diseases occurring include a continuous increase in carbapenem-resistant *Enterobacterales* (CRE) infections, which are healthcare-associated infections; a concentrated occurring of syphilis, a sexually transmitted infection; and a high proportion of imported infectious diseases. Malaria accounts for approximately 90% of all cases nationwide.

**③ What are implications?**

This analysis provides insights into infectious disease trends and characteristics in the Seoul Metropolitan Area, supporting the prevention and containment of outbreaks through prompt, on-site immediate responses based on regional collaboration.

## Introduction

Infectious diseases are a major international public health problem that constantly threatens our lives and safety. Having experienced Middle East respiratory syndrome (MERS) in 2015 and the coronavirus disease 2019 (COVID-19) pandemic from 2020 to 2024, the Korea Disease Control and Pre­vention Agency (KDCA) established five Regional Disease Response Centers across the nation in September 2020. Accordingly, each center serves as a regional public health base and hub, establishing a region-based infectious disease surveillance, preparedness, and response system.

The Capital Regional Center for Disease Control and Prevention (hereinafter referred to as the Capital Regional Center) has jurisdiction over four cities and provinces (Seoul Metropolitan City, Incheon Metropolitan City, Gyeonggi province, and Gangwon State), which account for 53.9% of total population of the Republic of Korea (ROK) [1]. Seoul, Gyeonggi, and Incheon are the nation’s largest population- and economic activity-dense regions, with a large volume of population movement crossing local government boundaries for commuting to work and school. Furthermore, because major international transport hubs including Incheon International Airport and Incheon Port are located here, management of the domestic introduction and initial spread of imported infectious diseases is extremely important. Northern Gyeonggi and northern Gangwon are border regions with North Korea, and the Gangwon region in particular has frequent mountainous, coastal tourism, and outdoor activities.

With the opening of the Infectious Disease Information System (IDIS) in January 2024, each Regional Disease Response Center oversees infectious disease notification tasks within its region, closely analyzes infectious disease surveillance status such as occurrence patterns and cluster outbreaks, and provides response results, including epidemiological investigations, as feedback to local governments. This study extracted a portion of the “2025 Infectious Disease Notification Status Annual Report” based on notification information from IDIS and analyzed the status and epidemiological characteristics of high-incidence and high-proportion notifiable infectious diseases under mandatory surveillance (Classes 1–3) that occurred in the Seoul Metropolitan Area during the year 2025. The purpose of this study is to identify trends in infectious disease occurrences in the Seoul Metropolitan Area through surveillance analysis data and utilize it as basic data to establish future policies for infectious disease prevention, management, preparedness, and response by the Capital Regional Center.

## Methods

Pursuant to Article 11 of the “Infectious Disease Control and Prevention Act”, physicians and other medical personnel must report patients with infectious diseases, infectious disease pathogen carriers, or suspected cases; Class 1 notifiable infectious diseases must be reported immediately, and Class 2 and 3 notifiable infectious diseases must be reported within 24 hours. Based on the notification data (centered on 2025) of patients with notifiable infectious diseases in the Seoul Metropolitan Area (Seoul, Incheon, Gyeonggi, Gangwon) collected through IDIS, the authors analyzed notification counts and proportions as frequencies and percentages (%), respectively, excluding duplicates, for 66 out of a total of 67 notifiable infectious diseases under mandatory surveillance in Classes 1, 2, and 3, excluding acquired immunodeficiency syndrome (AIDS). In this study, Class 4 notifiable infectious diseases (23 diseases), which are subjects of sentinel surveillance, were excluded. In addition, further analysis was conducted on suspected case reports of Class 1 notifiable infectious diseases and cases classified as probable cases received through the KDCA 1339 Call Center or public health centers. Statistical analysis was performed using Microsoft Excel 2024.

## Results

Among the notifiable infectious diseases that occurred in the Seoul Metropolitan Area during the year 2025, excluding AIDS, 39 out of 66 notifiable infectious diseases under mandatory surveillance (Classes 1–3) were reported, with a total of 74,895 cases. This figure represents an 18.9% decrease compared to 2024 (38 diseases reported, 92,340 total cases). In 2025, a total of 508 suspected case notifications for Class 1 notifiable infectious diseases were identified in the Seoul Metropolitan Area; among these, 72 were probable cases, and there were no confirmed cases. This accounts for 66.4% (508/765 cases) of the total nationwide suspected case responses. By infectious disease, MERS accounted for 350 cases (68.9%), human infection with zoonotic influenza for 107 cases (21.1%), Marburg virus disease for 19 cases (3.7%), Crimean-Congo hemorrhagic fever for 10 cases (2.0%), and Ebola virus disease for 9 cases (1.8%); Nipah virus disease, which was added as a Class 1 notifiable infectious disease as of September 8, 2025 for 7 cases (1.4%); plague and diphtheria had 2 cases each, and smallpox and tularemia had 1 case each. Otherwise, there were no suspected case notifications for Lassa fever, anthrax or botulism (Table 1). By region, Gyeonggi accounted for 245 cases (48.2%), Seoul for 186 cases (36.6%), Incheon for 52 cases (10.2%), and Gangwon for 25 cases (4.9%) (Table 2). The total number of Class 2 and Class 3 notifiable infectious disease patients (including probable cases, excluding AIDS) in the Seoul Metropolitan Area was 74,895, accounting for 54.0% of all cases nationwide (138,709). Healthcare-associated infectious diseases such as carbapenem-resistant *Enterobacterales* (CRE) infections were the highest at 31,114 cases (63.4% of 49,055 nationwide), followed by vaccine-preventable diseases such as varicella, mumps, pertussis, and measles at 20,842 cases (48.5% of 43,002 nationwide), and tuberculosis at 8,216 cases (48.1% of 17,070 nationwide) (Figure 1). This accounts for 80.3% (60,172 cases) of all reported infectious disease occurrences in the Seoul Metropolitan Area.

The high-incidence infectious diseases in the Seoul Metro­politan Area in 2025 (Table 3) were CRE infection, varicella, and tuberculosis, in that order; CRE infection reached 31,113 cases, continuing an upward trend every year and representing the highest figure since statistics began in 2017. Varicella recorded 14,904 cases, the highest incidence in the past 5 years, and has been on an increasing trend every year since 2023 following a sharp decline during the COVID-19 pandemic. Tuberculosis had 8,216 cases, showing a decreasing trend every year since 2022, a 6.2% decrease compared to the previous year (8,763 cases). Gyeonggi had the highest number of cases at 3,732, and also showed the largest decrease compared with the previous year at 8.4%.

Among infectious diseases with a cumulative total of 10 or more cases in 2025, high-proportion infectious diseases with a high proportion of occurrences in the Seoul Metropolitan Area relative to the nationwide total were Mpox, malaria, and Lyme disease, in that order. For Mpox, the Seoul Metropolitan Area accounted for the highest proportion, with 25 out of 27 nationwide cases (92.6%). For malaria, the Seoul Metropolitan Area accounted for 89.5% with 538 out of 601 nationwide cases, with Gyeonggi having the highest number at 332 cases, followed by Incheon, Seoul, and Gangwon. For Lyme disease, the Seoul Metropolitan Area showed a high proportion of 70.7%, with 29 out of 41 nationwide cases. In addition, infectious diseases with a low proportion of occurrence in the Seoul Metropolitan Area of less than 50% were scrub typhus, hemorrhagic fever with renal syndrome (HFRS), and severe fever with thrombocytopenia syndrome (SFTS), in that order. Scrub typhus had the lowest proportion at 15.0%, with 510 Seoul Metropolitan Area cases out of 3,405 nationwide cases, while HFRS accounted for 20.9% with 48 Seoul Metropolitan Area cases out of 230 nationwide cases, and SFTS accounted for 32.1% with 90 Seoul Metropolitan Area cases out of 280 nationwide cases.

In 2025, the infectious diseases (cumulative total of 10 or more cases) that increased the most compared with the previous year in the Seoul Metropolitan Area were enterohemorrhagic *Escherichia coli* (EHEC) infection, measles, and Q fever, in that order. EHEC infection occurred in 301 cases, an increase of 161.7% compared with the previous year (115 cases). In particular, many cases occurred during the summer season from June to August, and by region, a large number of cases occurred in Gyeonggi, Seoul, Incheon, and Gangwon, in that order. Measles occurred in 37 cases, an increase of 117.6% compared with the previous year (17 cases). The infection routes of confirmed cases were identified as imported cases or cases related to imported cases. By region, most cases occurred in Seoul (17 cases) and Gyeonggi (16 cases), and by age group, they were most common in their 30s (15 cases), 40s (7 cases), and 20s (6 cases), in that order, with the proportion of adults aged 20 and older (30 cases) being high at 81.1%. Q fever occurred in 32 cases, an increase of 88.2% compared with the previous year (17 cases). In particular, patients increased significantly in Gyeonggi, with Gyeonggi accounting for 81.3% of the occurrences in the Seoul Metropolitan Area. The infectious disease with the largest decrease compared with the previous year was pertussis, with 2,133 cases occurring in 2025, representing a 91.9% decrease compared with the previous year (26,247 cases). Pertussis showed the largest epidemic in the ROK in 2024 and was found to have decreased in 2025. By city and province, the largest number of cases occurred in Gyeonggi, followed by Seoul, Incheon, and Gangwon.

## Discussion

In 2025, the number of reported cases of notifiable infectious diseases under mandatory surveillance in the Seoul Metropolitan Area totaled 74,895 (excluding AIDS), accounting for 54.0% of the national total of 138,709 cases. There were a total of 508 suspected case reports for Class 1 notifiable infectious diseases (18 diseases), among which 72 were probable cases, and there were no confirmed cases. A total of 69,118 cases were reported for Class 2 notifiable infectious diseases (21 diseases), and a total of 5,777 cases were reported for Class 3 notifiable infectious diseases (27 diseases, excluding AIDS).

The Capital Regional Center manages priority cases of high-risk groups and cluster occurrences, as well as cluster cases of major infectious diseases, considering the risk level and characteristics of each infectious disease. For measles, among the reported cases (including probable cases), 37 cases were in the Seoul Metropolitan Area, meaning the Capital Regional Center responded to 47.4% of the total cases (78 cases), and all infection routes were imported or imported-related cases. According to the World Health Organization (WHO), as of the end of 2025 (global measles vaccination coverage), the coverage for the first dose of measles-containing vaccine (MCV1) by 2 years of age was 84% and the second dose (MCV2) was 77%, falling significantly short of the 95% level for 2 doses required to interrupt measles transmission [2]. Conversely, the ROK’s MMR coverage at 2 years of age was high at 96.7%, and MMR coverage at 6 years of age was high at 95.6% [3]. Amid increasing global measles incidence due to increased international exchange and travel following COVID-19, imported cases continue to occur, and a response system is operational to achieve early detection and block spread, including mandatory surveillance of reported cases and technical support for epidemiological investigations. A total of 114 tuberculosis priority cases were managed as priority cases due to infant and young child tuberculosis exposure, additional case occurrences, multidrug-resistant smear positivity, etc. For CRE infections, including pathogen carriers, there were 31,113 cases in the Seoul Metropolitan Area, and the Capital Regional Center responded to 63.4% of the total cases (49,053 cases). There were a total of 25 malaria cluster events (2 or more cases), and case occurrence surveillance as well as response to all cluster events are being conducted jointly by the Center and local governments. Legionellosis is showing an increasing trend in notified cases due to the effects of an aging population, aging buildings, and climate change; in 2025, the total number of notified patients in the Seoul Metropolitan Area was 396, an increase of 43.5% compared with the previous year (276 patients), accounting for 61.9% of the national total (640 patients). The Capital Regional Center is managing and responding to cases on a case-by-case basis, particularly regarding occurrences of patients within medical institutions, through joint public-private epidemiological investigations, expert advisory meetings, intervention measures, and environmental surveillance.

As a result of analyzing the trends of infectious diseases occurring in the Seoul Metropolitan Area, the following characteristics were identified.

First, there is a continuous increase in CRE infection, a healthcare-associated infectious disease. In the Seoul Metro­politan Area, there are 25 advanced general hospitals (out of 47 nationwide) and 700 long-term care hospitals (out of 1,306 nationwide), with the number of medical institutions accounting for more than 50% of the national total; recently, cases of CRE infection occurred in 23,847 patients in 2023, 26,623 in 2024, and 31,113 in 2025, showing an average growth rate of 17.7% compared with the previous year. This is because domestic CRE infection requires notifications for pathogen carriers as well as patients, and notified cases of pathogen carriers are increasing due to the recent active implementation of preemptive testing by medical institutions; it is expected that the nationwide increasing trend will continue for the time being [4]. The Capital Regional Center is implementing response measures for healthcare-associated infection prevention and management, such as conducting on-site inspections and strengthening education for medical institutions under its jurisdiction.

Second, the occurrence of syphilis, a sexually transmitted infection, is concentrated. Syphilis was converted to a mandatory surveillance system (Class 3 notifiable infectious disease) starting in 2024, and 1,630 cases were notified in the Seoul Metropolitan Area (out of 2,790 nationwide). In 2025, the total number of notified syphilis patients in the Seoul Metropolitan Area was 1,357, a slight decrease compared with the previous year, but it accounted for 61.4% of the national total (2,211 patients); in particular, the incidence proportion was high in Seoul and Gyeonggi, where there are many young people with frequent sexual contact. In addition, Mpox—which is not classified as a sexually transmitted infection among notifiable infectious diseases but mainly occurs through sexual contact—saw all 4 cases occur in the Seoul Metropolitan Area including the first imported case in the country in 2022, went through a nationwide outbreak in 2023 (151 patients total, 122 in the Seoul Metropolitan Area), showed sporadic occurrences in 2024 (17 nationwide, 16 in the Seoul Metropolitan Area), and following the lifting of the Public Health Emergency of International Concern by the WHO in September 2025, the ROK shifted to an integrated management system for Clade Ib and IIb in December and has been responding under this system. In 2025, 25 cases were notified in the Seoul Metropolitan Area, accounting for a high proportion of 92.6% of the national total (27 cases). All Mpox patients notified in the ROK up to 2025 were Clade IIb. AIDS was excluded from this study because, although its primary transmission route is sexual contact, the KDCA manages the national notification status through the HIV/AIDS Supporting Network System (HASNet). Going forward, there is a need to further strengthen awareness improvement, medical accessibility enhancement, and education and promotion of preventive measures for sexually transmitted infections.

Third, the Seoul Metropolitan Area accounts for a high proportion of imported infectious disease cases (Supplementary Table 1; available online). According to imported infectious disease statistics from the Infectious Disease Portal [5], imported cases in the Seoul Metropolitan Area totaled 411 individuals in 2025, accounting for 64.9% of the national total (633 individuals). Infectious diseases with high numbers of imported cases in the Seoul Metropolitan Area were syphilis (138 cases), dengue fever (69 cases), and measles (32 cases), in that order; dengue fever, for which all domestic cases were imported, had 69 cases in the Seoul Metropolitan Area out of 110 cases nationwide, accounting for 62.7%; melioidosis showed a high proportion of 80.0%, with 4 out of 5 imported cases nationwide occurring in the Seoul Metropolitan Area; and all 3 cases (100%) of Zika virus infection were reported in the Seoul Metropolitan Area. For proactive prevention of imported infectious diseases, it is necessary to further improve public accessibility to campaigns targeting travelers departing the country.

Finally, for malaria, approximately 90% of national cases occur in the Seoul Metropolitan Area every year. The proportion of occurrences in the Seoul Metropolitan Area compared to the national total was 91.4% (683/747 cases) in 2023, 91.7% (654/713 cases) in 2024, and 89.5% (538/601 cases) in 2025, showing that most cases occur in the Seoul Metropolitan Area. This is because *Plasmodium vivax* malaria occurs mainly in northern Gyeonggi and some areas of Incheon in the ROK [4]. Lyme disease had 29 cases in the Seoul Metropolitan Area in 2025, accounting for 70.7% of the national total (41 cases), while SFTS showed a low proportion of 32.1%, with 90 cases in the Seoul Metropolitan Area out of the national total (280 cases). This is considered to be due to the influence of regional distribution differences, as both Lyme disease and SFTS are hard tick-borne infectious diseases, but SFTS is transmitted by *Haemaphysalis longicornis*, and *Haemaphysalis longicornis* is the absolute dominant species in the ROK, accounting for 78.46% of hard ticks [6].

Based on the “2025 Infectious Disease Notification Status Annual Report” in the ROK, as basic data, this study analyzed trends and characteristics of infectious diseases within the Seoul Metropolitan Area, focusing on descriptive statistics of the number of reported infectious disease occurrences (Classes 1 to 3) by provinces and cities under its jurisdiction. This study has public health significance in that it provides evidence for detecting outbreaks early, identifying high-risk areas and groups, and prioritizing preventive management projects and quarantine resources by understanding the scale, distribution, and trends of infectious disease occurrences in local communities. Going forward, the Capital Regional Center will strengthen cooperation systems with the four cities and provinces under its jurisdiction to prevent, manage, and rapidly respond to infectious diseases in the Seoul Metropolitanl Area, share common goals and strategies, and contribute to blocking the influx of infectious diseases and preventing further spread through immediate, field-centered response.

## Figures and Tables

**Figure 1:**
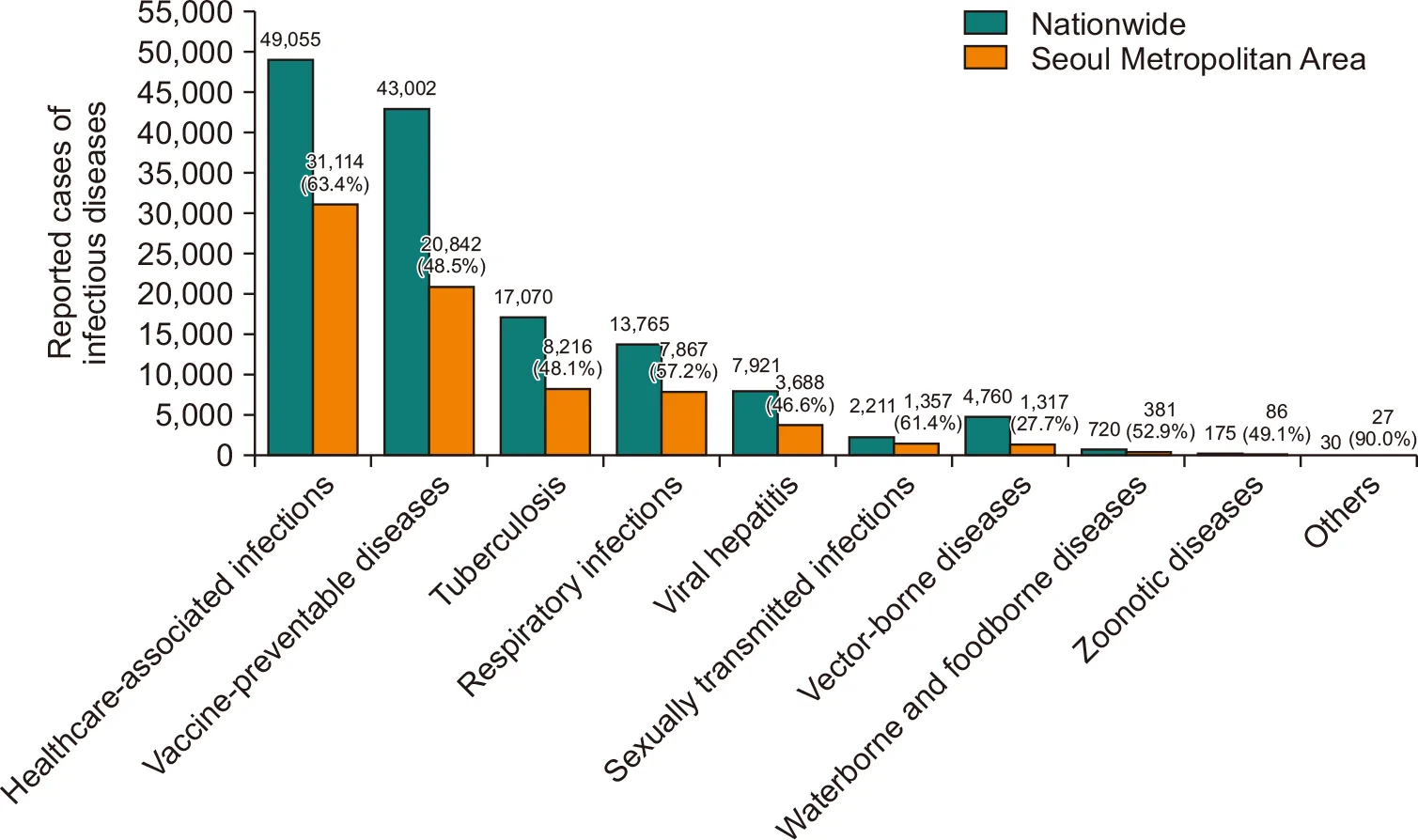
Status of reported cases of notifiable infectious diseases in the Seoul Metropolitan Area compared to that nationwide in 2025 Healthcare-associated infections: carbapenem-resistant *Enterobacterales* (CRE) infections, vancomycin-resistant *Staphylococcus aureus* (VRSA) infections. Vaccine-preventable diseases: chickenpox, mumps, pertussis, pneumococcal disease, measles, haemophilus influenzae type b, tetanus. Respiratory infections: scarlet fever, meningococcal disease, legionellosis. Viral hepatitis: hepatitis A, hepatitis E, hepatitis B, hepatitis C. Sexually transmitted infections: syphilis. Vector-borne diseases: malaria, scrub typhus, severe fever with thrombocytopenia syndrome (SFTS), dengue fever, hemorrhagic fever with renal syndrome, Lyme borreliosis, murine typhus, leptospirosis, Japanese encephalitis, chikungunya fever, melioidosis, Zika virus infection. Waterborne and foodborne diseases: enterohemorrhagic *Escherichia coli* (EHEC) infection, shigellosis, vibrio vulnificus sepsis, paratyphoid fever, typhoid fever. Zoonotic diseases: Creutzfeldt–Jakob disease (CJD), Q fever, brucellosis. Others: Hansen’s disease, monkeypox.

**Table 1:** Status of suspected cases of Class 1 notifiable infectious diseases in the Seoul Metropolitan Area (2024–2025)

Disease	2025				2024		
	Suspected casesa)	Probable casesb)	(Percentage)		Suspected casesa)	Probable casesb)	(Percentage)
Total	508	72	(14.2)		511	44	(8.6)
Ebola virus disease	9	2	(22.2)		4	0	(0.0)
Marburg hemorrhagic fever	19	4	(21.1)		12	0	(0.0)
Lassa fever	-	-	-		1	0	(0.0)
Crimean-Congo hemorrhagic fever	10	3	(30.0)		1	0	(0.0)
South American hemorrhagic fever	-	-	-		-	-	-
Rift Valley fever	-	-	-		6	0	(0.0)
Smallpox	1	0	(0.0)		-	-	-
Plague	2	0	(0.0)		80	1	(1.3)
Anthrax	-	-	-		-	-	-
Botulism	-	-	-		-	-	-
Tularemia	1	1	(100.0)		-	-	-
SARS	-	-	-		-	-	-
MERS	350	42	(12.0)		366	38	(10.4)
Diphtheria	2	2	(100.0)		-	-	-
Human infection with zoonotic influenza	107	18	(16.8)		41	5	(12.2)
Novel influenza	-	-	-		-	-	-
Emerging infectious diseases	-	-	-		-	-	-
Nipah virus diseasec)	7	0	(0.0)		-	-	-

Unit: Case, %. No confirmed cases. SARS=severe acute respiratory syndrome; MERS=Middle East respiratory syndrome. ^a)^Suspected reports: the number of suspected cases reported by public health centers, medical institutions, the 1339 hotline, quarantine stations, etc. ^b)^Probable cases: the number of cases classified based on clinical symptoms and epidemiological associations and referred for diagnostic testing. ^c)^Addi­tionally designated on Sep. 8, 2025.

**Table 2:** Status of suspected cases of Class 1 notifiable infectious diseases in the Seoul Metropolitan Area, by province in 2024–2025

Classification		Total	Seoul	Incheon	Gyeonggi	Gangwon
2025	Suspected cases	508	186	52	245	25
	(Per 1,000,000 population)	(18.4)	(19.8)	(17.2)	(18.0)	(16.6)
	Probable cases	72	20	6	33	13
	Percentage	(14.2)	(10.8)	(11.5)	(13.5)	(52.0)
2024	Suspected cases	511	177	38	286	10
	(Per 1,000,000 population)	(18.4)	(18.9)	(12.6)	(21.0)	(6.6)
	Probable cases	44	9	5	26	4
	Percentage	(8.6)	(5.1)	(13.2)	(9.1)	(40.0)

Unit: Case, incidence rate (per 1,000,000 population), %.

**Table 3:** National notifiable infectious diseases surveillance, by province–Seoul Metropolitan Area in 2025

Class	Disease		No. of notifications														
			Nationwide	Seoul Metropolitan Area	Proportion	Seoul			Incheon			Gyeonggi			Gangwon		Seoul Metropolitan Area in 2024
						Case	Incidence rate		Case	Incidence rate		Case	Incidence rate		Case	Incidence rate	
Class 2	Tuberculosis		17,070	8,216	48.1	2,853	30.9		928	30.7		3,732	27.3		703	46.6	8,763
	Varicella		30,248	14,904	49.3	3,592	38.56		1,293	42.58		8,966	65.39		1,053	69.59	14,567
	Measles		78	37	47.4	17	0.18		2	0.07		16	0.12		2	0.13	17
	Typhoid fever		33	8	24.2	1	0.01		1	0.03		6	0.04		-	0.00	20
	Paratyphoid fever		24	12	50.0	7	0.08		-	0.00		3	0.02		2	0.13	9
	Shigellosis		64	32	50.0	19	0.20		2	0.07		11	0.08		-	0.00	24
	EHEC infection		531	301	56.7	95	1.02		37	1.22		161	1.17		8	0.53	115
	Viral hepatitis A		1,141	588	51.5	183	1.96		63	2.07		312	2.28		30	1.98	638
	Pertussis		5,491	2,133	38.8	486	5.22		256	8.43		1,195	8.71		196	12.95	26,247
	Mumps		6,720	3,494	52.0	890	9.55		377	12.42		1,981	14.45		246	16.26	3,323
	Meningococcal disease		12	6	50.0	1	0.01		-	0.00		5	0.04		-	0.00	15
	Haemophilus influenzae Type b		1	-	-	-	0.00		-	0.00		-	0.00		-	0.00	-
	Pneumococcal disease		440	266	60.5	106	1.14		27	0.89		120	0.88		13	0.86	276
	Hansen’s disease		3	2	66.7	-	0.00		-	0.00		2	0.01		-	0.00	2
	Scarlet fever		13,113	7,465	56.9	1,684	18.08		1,173	38.63		4,152	30.28		456	30.14	4,532
	VRSA infection		2	1	50.0	-	0.00		1	0.00		-	0.00		-	0.00	1
	CRE infection		49,053	31,113	63.4	12,553	134.75		3,887	128.01		13,464	98.19		1,209	79.90	26,623
	Viral hepatitis E		915	540	59.0	183	1.96		37	1.22		277	2.02		43	2.84	449
Class 3	Tetanus		24	8	33.3	1	0.01		2	0.07		5	0.04		-	0.00	17
	Viral hepatitis B (acute)		226	103	45.6	33	0.35		11	0.36		53	0.39		6	0.40	125
	Japanese encephalitis		7	5	71.4	1	0.01		-	0.00		3	0.02		1	0.07	14
	Viral hepatitis C		5,639	2,457	43.6	790	8.48		472	15.54		1,076	7.85		119	7.86	2,865
	Malaria		601	538	89.5	70	0.75		108	3.56		332	2.42		28	1.85	654
	Legionellosis		640	396	61.9	168	1.80		35	1.15		177	1.29		16	1.06	276
	Vibrio vulnificus sepsis		68	28	41.2	9	0.10		5	0.16		13	0.09		1	0.07	30
	Murine typhus		16	9	56.3	1	0.01		5	0.16		3	0.02		-	0.00	28
	Scrub typhus		3,405	510	15.0	105	1.13		56	1.84		330	2.41		19	1.26	701
	Leptospirosis		53	7	13.2	1	0.01		-	0.00		6	0.04		-	0.00	8
	Brucellosis		4	1	25.0	-	0.00		-	0.00		1	0.01		-	0.00	1
	HFRS		230	48	20.9	8	0.09		9	0.30		27	0.20		4	0.26	58
	Creutzfeldt–Jakob disease		99	53	53.5	22	-		7	-		22	-		2	-	43
	Dengue fever		110	69	62.7	27	0.24		11	0.23		29	0.16		2	0.13	137
	Q fever		72	32	44.4	3	0.29		1	0.36		26	0.21		2	0.13	17
	Lyme borreliosis		41	29	70.7	5	0.03		-	0.03		23	0.19		1	0.13	17
	Melioidosis		5	4	80.0	2	0.05		-	0.00		1	0.17		1	0.07	1
	Chikungunya fever		9	5	55.6	4	0.02		1	0.00		-	0.01		-	0.07	7
	SFTS		280	90	32.1	14	0.04		3	0.03		42	0.00		31	0.00	74
	Zika virus infection		3	3	100.0	1	0.15		-	0.10		2	0.31		-	2.05	-
	Syphilis	Primary	721	440	61.0	189	0.01		60	0.00		177	0.01		14	0.00	569
		Secondary	449	290	64.6	144	2.03		38	1.98		105	1.29		3	0.93	334
		Tertiary	50	32	64.0	7	1.55		3	1.25		22	0.77		-	0.20	29
		Congenital	12	8	66.7	5	0.08		2	0.10		1	0.16		-	0.00	3
		(Early) latent	979	587	60.0	236	0.05		85	0.07		248	0.01		18	0.00	695
	Monkeypox		27	25	92.6	14	2.53		2	2.80		9	1.81		-	1.19	16

Unit: Case, %, incidence rate (per 100,000 population). No. of cases contain all case classifications of the disease (confirmed, suspected, asymptomatic carrier), respectively. Classified by the patient’s address, except for VRSA and CRE, which are classified by the medical institution’s location; military malaria cases are classified by the managing public health center’s location. Only infectious diseases reported in 2025 were included. AIDS statistics by region are not provided to protect personal information and prevent regional bias. EHEC=enterohemorrhagic *Escherichia coli*; VRSA=vancomycin-resistant *Staphylococcus aureus*; CRE, carbapenem-resistant *Enterobacterales*; HFRS=hemorrhagic fever with renal syndrome; SFTS=severe fever with thrombocytopenia syndrome; AIDS=acquired immune deficiency syndrome.
