# Supplementary material for: Status of Notifiable Infectious Diseases Reporting in the Seoul Metropolitan Area, Republic of Korea, 2025
Source: Public Health Wkly Rep. 2026 Nov 12;19(43):1838–57. doi: 10.56786/PHWR.2026.19.43.4 (PMC13759229; doi:10.56786/PHWR.2026.19.43.4)
Supplement: Supplementary file 1 [file phwr-19-43-1838-supple.pdf]

보충 표 1. 2025년 수도권역 법정 감염병 해외유입 현황(시도별)

구분		전국	수도권역	비율	서울	인천	경기	강원
총계		633	411	64.9	156	48	192	15
2급	수두	35	19	54.3	8	0	11	0
	홍역	55	32	58.2	15	1	14	2
	장티푸스	5	1	20.0	0	1	0	0
	파라티푸스	2	1	50.0	1	0	0	0
	세균성이질	20	13	65.0	8	1	4	0
	장출혈성대장균(EHEC) 감염증	21	10	47.6	7	1	2	0
	A형간염	31	19	61.3	3	3	13	0
	백일해	23	14	60.9	3	0	11	0
	유행성이하선염	6	3	50.0	1	0	2	0
	한센병	3	2	66.7	0	0	2	0
	성홍열	4	2	50.0	0	0	2	0
	카바페넴내성장내세균목 (CRE) 감염증	2	2	100.0	1	1	0	0
	E형간염	12	9	75.0	4	0	5	0
3급	B형간염	1	1	100.0	0	0	1	0
	C형간염	31	19	61.3	5	3	11	0
	말라리아	56	26	46.4	8	5	11	2
	레지오넬라증	6	6	100.0	2	1	2	1
	쯔쯔가무시증	6	2	33.3	1	0	1	0
	렙토스피라증	1	0	0.0	0	0	0	0
	신증후군출혈열	1	0	0.0	0	0	0	0
	뎅기열	110	69	62.7	27	11	29	2
	라임병	8	7	87.5	2	0	5	0
	유비저	5	4	80.0	2	0	1	1
	치쿤구니야열	9	5	55.6	4	1	0	0
	지카바이러스감염증	3	3	100.0	1	0	2	0
	매독	173	138	79.8	50	18	63	7
	엠폭스	4	4	100.0	3	1	0	0

단위: 명, %.

Supplementary Table 1. National notifiable diseases for imported cases, by province–Seoul Metropolitan Area in 2025

Classification		Nation wide	Seoul Metropolitan Area	Proportion	Seoul	Incheon	Gyeonggi	Gangwon
Total		633	411	64.9	156	48	192	15
Class 2	Varicella	35	19	54.3	8	0	11	0
	Measles	55	32	58.2	15	1	14	2
	Typhoid fever	5	1	20.0	0	1	0	0
	Paratyphoid fever	2	1	50.0	1	0	0	0
	Shigellosis	20	13	65.0	8	1	4	0
	EHEC	21	10	47.6	7	1	2	0
	Viral hepatitis A	31	19	61.3	3	3	13	0
	Pertussis	23	14	60.9	3	0	11	0
	Mumps	6	3	50.0	1	0	2	0
	Hansen's disease	3	2	66.7	0	0	2	0
	Scarlet fever	4	2	50.0	0	0	2	0
	CRE infection	2	2	100.0	1	1	0	0
	Viral Hepatitis E	12	9	75.0	4	0	5	0
Class 3	Viral hepatitis B	1	1	100.0	0	0	1	0
	Viral hepatitis C	31	19	61.3	5	3	11	0
	Malaria	56	26	46.4	8	5	11	2
	Legionellosis	6	6	100.0	2	1	2	1
	Scrub typhus	6	2	33.3	1	0	1	0
	Leptospirosis	1	0	0.0	0	0	0	0
	Hemorrhagic fever with renal syndrome	1	0	0.0	0	0	0	0
	Dengue fever	110	69	62.7	27	11	29	2
	Lyme Borreliosis	8	7	87.5	2	0	5	0
	Melioidosis	5	4	80.0	2	0	1	1
	Chikungunya fever	9	5	55.6	4	1	0	0
	Zika virus infection	3	3	100.0	1	0	2	0
	Syphilis	173	138	79.8	50	18	63	7
	Monkeypox	4	4	100.0	3	1	0	0

Unit: case, %. EHEC=enterohemorrhagic *Escherichia coli*; CRE=carbapenem-resistant *Enterobacterales*.
