# Supplementary material for: Status of Notifiable Infectious Diseases Reporting in the Seoul Metropolitan Area, Republic of Korea, 2025
Source: Public Health Wkly Rep. 2026 Nov 12;19(43):1838–57. doi: 10.56786/PHWR.2026.19.43.4 (PMC13759229; doi:10.56786/PHWR.2026.19.43.4)
Supplement: Translation

# Translation: 2025년 수도권역 법정 감염병 신고 현황 분석

**Authors:** 성남 김, 지혜 추, 현정 김, 미정 고, 종희 김

**Affiliations:** 질병관리청 수도권질병대응센터 감염병대응과

**Keywords:** 감염병, 통계, 공중보건, 수도권질병대응센터

## Abstract

**목적:**

수도권질병대응센터(수도권센터)는 우리나라 전체 인구의 53.9%가 속해 있는 4개 시도(서울특별시, 인천광역시, 경기도, 강원특별자치도)를 관할한다. 본 연구는 방역통합정보시스템 신고정보를 기반으로 하여 2025년 한 해 동안 수도권역에서 발생한 전수감시 법정 감염병(제1–3급)의 다발생‧고비중 현황 및 특성을 분석하였다. 본 연구는 감시분석자료를 통해 수도권역 내 감염병 발생의 경향성을 파악하여, 향후 수도권센터의 감염병 예방‧관리 및 대비‧대응 정책을 수립하는 기초자료로 활용하는 것을 목적으로 한다.

**방법:**

방역통합정보시스템을 통해 수집한 수도권역 법정 감염병 환자의 발생 신고자료를 토대로 제1‧2‧3급 전수감시 감염병 총 66종을 대상으로 중복을 제외하고 발생 신고건수, 신고비중을 각각 빈도수와 백분율(%)로 분석하였다.

**결과:**

2025년 한 해 동안 수도권역에서 발생한 법정 감염병 중 전수감시 감염병의 발생 신고사례는 총 74,895명(후천성면역결핍증 제외)으로 2024년(총 92,340명)보다 18.9% 감소했다. 다발생 신고 감염병은 카바페넴내성장내세균목(carbapenem-resistant *Enterobacterales*, CRE) 감염증, 수두, 결핵 순이며, 전국 대비 수도권역 발생 비중이 높은 감염병은 엠폭스, 말라리아, 라임병 순이었다. 또한 발생 비중이 낮은 감염병은 쯔쯔가무시증, 신증후군출혈열, 중증열성혈소판감소증후군 순으로 나타났다. 2025년 수도권역에서 전년 대비 가장 증가한 감염병은 장출혈성대장균 감염증, 홍역, 큐열 순이다. 전년 대비 가장 감소폭이 큰 감염병은 백일해였다.

**결론:**

수도권역에서 발생하는 감염병의 특성은 의료관련감염병인 CRE 감염증의 지속적인 증가, 성매개감염병인 매독 발생 집중, 해외유입 감염병의 수도권역 발생 비중이 높다는 것이다. 또한 말라리아는 매년 전국 발생의 약 90%가 수도권역에서 발생하고 있다. 수도권역에서 발생하는 감염병의 경향과 특성을 파악하고 감염병 예방‧관리 및 대비‧대응 정책을 수립하는 데 본 연구를 근거자료로 활용할 수 있을 것이다. 수도권센터는 수도권역의 감염병 발생 대응을 위해 관할 4개 시도와 협력체계를 강화하고 현장 중심의 즉각적인 대응으로 감염병의 유입을 차단하고 추가 확산 방지에 기여할 것이다.

**핵심요약**

**① 이전에 알려진 내용은?**

수도권질병대응센터는 4개 시도(서울특별시, 인천광역시, 경기도, 강원특별자치도)를 관할하며 감염병 발생 감시 및 즉각 대응체계를 구축하였다.

**② 새로이 알게 된 내용은?**

2025년 한 해 동안 수도권역에서 발생한 법정 감염병 중 전수감시 감염병의 발생 신고는 총 74,895명(후천성면역결핍증 제외)으로 2024년(총 92,340명) 대비 18.9% 감소했다. 수도권역에서 발생하는 감염병의 특성은 의료관련감염병인 카바페넴내성장내세균목 감염증의 지속적인 증가, 성매개감염병인 매독 발생 집중, 해외유입 감염병의 수도권역 발생 비중이 높다는 것이다. 말라리아는 전국 발생의 약 90%를 차지한다.

**③ 시사점은?**

본 감시분석은 수도권역 내 감염병 발생의 경향과 특성을 이해하는 데 의의가 있다. 수도권질병대응센터는 본 분석을 바탕으로 감염병 정책전략을 수립하고, 지역 협업 기반의 현장 중심 대응을 통해 감염병의 유입 차단 및 확산 방지에 기여하고자 한다.

## 서 론

감염병은 끊임없이 우리의 생명과 안전을 위협하는 중대한 국제적 보건 문제이다. 2015년 중동호흡기증후군(Middle East respiratory syndrome, MERS)과 2020년부터 2024년까지 코로나바이러스감염증-19(코로나19) 유행을 겪으며 질병관리청은 2020년 9월 전국에 5개 권역질병대응센터를 설치했다. 이에 각 센터는 지역 공중보건의 거점과 허브 역할을 수행하며 권역 기반의 감염병 발생 감시와 대비‧대응체계를 구축하였다.

수도권질병대응센터(수도권센터)는 우리나라 전체 인구의 53.9%가 속해 있는 4개 시도(서울특별시[서울], 인천광역시[인천], 경기도[경기], 강원특별자치도[강원])를 관할한다[1]. 서울‧경기‧인천은 국내 최대 인구 및 경제활동 밀집지역이며, 출퇴근‧통학 등으로 지자체 경계를 넘는 인구 이동량이 많다. 또한 인천국제공항과 인천항을 비롯한 주요 국제교통 거점이 위치해 있어서 해외유입 감염병의 국내 유입과 초기 확산 관리가 매우 중요하다. 경기 북부와 강원 북부지역은 북한과의 접경지역이며, 특히 강원지역은 산악‧해안관광‧야외활동이 많은 지역이다.

2024년 1월 방역통합정보시스템이 개통되면서 각 질병대응센터는 권역 내 감염병 신고업무를 소관하며 발생 양상 및 집단 유행 등 감염병 감시 현황을 면밀하게 분석하고, 역학조사 등 대응 결과를 지자체에 환류하고 있다. 본 연구는 방역통합정보시스템 신고정보를 기반으로 한 「2025 감염병 신고 현황 연보」의 일부를 발췌하여 2025년 한 해 동안 수도권역에서 발생한 전수감시 법정 감염병(제1–3급)의 다발생‧고비중 감염병 현황 및 역학적 특성을 분석하였다. 본 연구는 감시분석자료를 통해 수도권역 내 감염병 발생의 경향성을 파악하여, 향후 수도권센터의 감염병 예방‧관리 및 대비‧대응 정책을 수립하는 기초자료로 활용하는 것을 목적으로 한다.

## 방 법

「감염병의 예방 및 관리에 관한 법률」 제11조에 근거하여 의사 등은 감염병 환자 또는 감염병 병원체 보유자 또는 감염병 의심환자 등을 신고하며, 제1급 감염병은 즉시, 제2‧3급 감염병은 24시간 이내에 신고하도록 하고 있다. 본 연구는 방역통합정보시스템을 통해 수집된 수도권역(서울, 인천, 경기, 강원) 내 법정 감염병 환자의 발생 신고자료(2025년 중심)를 토대로 제1‧2‧3급 전수감시 감염병 총 67종 중 후천성면역결핍증(acquired immune deficiency syndrome, AIDS)을 제외한 66종을 대상으로 중복을 제외하고 발생 신고건수, 신고비중을 각각 빈도수와 백분율(%)로 분석하였다. 본 연구에서 표본감시 대상 감염병인 제4급 감염병(23종)은 제외하였다. 또한 질병관리청 1339콜센터 또는 보건소를 통해 접수된 제1급 감염병 의심신고건 및 의사환자 분류 사례를 추가 분석하였다. 통계 분석은 Microsoft Excel 2024를 활용하였다.

## 결 과

2025년 한 해 동안 수도권역에서 발생한 법정 감염병 중 AIDS를 제외하고, 전수감시 감염병(제1–3급) 66종 중 39종이 신고되었고, 발생 사례는 총 74,895명이었다. 이는 2024년(38종 신고, 총 92,340건)보다 18.9% 감소한 수치이다. 2025년 수도권역에서 확인된 제1급 감염병 의심신고 사례는 총 508건이며 이 중 의사환자 사례는 72건이었고, 확진 사례는 없었다. 이는 전국 총 의심환자 대응건수의 66.4% (508/765건)에 해당한다. 감염병별로는 MERS가 350건(68.9%), 동물인플루엔자 인체감염증 107건(21.1%), 마버그열 19건(3.7%), 크리미안콩고출혈열 10건(2.0%), 에볼라바이러스병 9건(1.8％) 순이었고, 2025년 9월 8일부터 제1급 감염병으로 추가 지정된 니파바이러스감염증 의심신고는 7건(1.4％)이었다(표 1). 페스트와 디프테리아는 각 2건, 두창과 야토병이 각 1건, 이외 라싸열, 탄저, 보툴리눔독소증 등은 의심신고가 없었다(표 1). 지역별로는 경기 245건(48.2%), 서울 186건(36.6%), 인천 52건(10.2%), 강원 25건(4.9%) 순이었다(표 2). 수도권역 제2‧3급 감염병 환자(의사환자 포함, AIDS 제외)는 총 74,895명으로, 전국 환자(138,709명)의 54.0%를 차지한다. 카바페넴내성장내세균목(carbapenem-resistant *Enterobacterales*, CRE) 감염증 등 의료관련감염병 31,114명(전국 49,055명의 63.4%), 수두, 유행성이하선염, 백일해, 홍역 등 예방접종대상감염병 20,842명(전국 43,002명의 48.5%), 결핵 8,216명(전국 17,070명의 48.1%) 순으로 높게 나타났다(그림 1). 이는 수도권역 전체 발생 신고 감염병의 80.3% (60,172명)를 차지한다.

2025년 수도권역에서 다수 발생한 감염병은 CRE 감염증, 수두, 결핵 순으로, CRE 감염증은 31,113명으로 매년 증가 추세를 이어가며 2017년부터 집계한 이후 가장 높은 수치를 나타냈다(표 3). 수두는 14,904명으로 최근 5년 중 최다 발생하였고, 코로나19 대유행을 거치며 급감 후 2023년부터 매년 증가 추세에 있다. 결핵은 8,216명 발생했고 2022년부터 매년 감소 추세에 있으며, 전년(8,763명) 대비 6.2% 감소했다. 경기가 3,732명으로 가장 많이 발생했으며, 전년 대비 감소 폭도 8.4%로 가장 높게 나타났다.

2025년 누적 10명 이상 발생한 감염병 중 전국 대비 수도권역 발생 비율이 높은 고비중 감염병은 엠폭스, 말라리아, 라임병 순으로 나타났다. 엠폭스는 전국 27명 중 수도권역이 25명(92.6%)으로 가장 높은 비중을 차지했다. 말라리아는 전국 601명 중 수도권역이 538명으로 89.5%를 차지하며, 경기가 332명으로 가장 많이 발생했고 그 다음 인천, 서울, 강원 순이었다. 라임병은 전국 41명 중 수도권역이 29명으로 70.7%로 높은 비중을 보였다. 또한 수도권역 발생 비중이 50% 미만으로 낮은 감염병은 쯔쯔가무시증, 신증후군출혈열, 중증열성혈소판감소증후군(severe fever with thrombocytopenia syndrome, SFTS) 순이었다. 쯔쯔가무시증은 전국 3,405명 중 수도권역 510명으로 15.0%의 가장 낮은 비중이었으며, 신증후군출혈열은 전국 230명 중 수도권역 48명으로 20.9%, SFTS는 전국 280명 중 수도권역 90명으로 32.1%로 나타났다.

2025년 수도권역에서 전년 대비 발생(누적 10명 이상)이 가장 많이 증가한 감염병은 장출혈성대장균 감염증, 홍역, 큐열 순이다. 장출혈성대장균 감염증은 301명 발생했고, 전년(115명) 대비 161.7% 증가했다. 특히 여름철인 6월부터 8월에 환자가 다수 발생했으며, 시도별로는 경기, 서울, 인천, 강원 순으로 많은 환자가 발생했다. 홍역은 37명 발생했고, 전년(17명) 대비 117.6% 증가했다. 확진자의 감염경로는 해외유입 또는 해외유입 관련 사례로 확인되었다. 시도별로는 서울(17명)과 경기(16명)에서 대부분의 환자가 발생했고, 연령대별로는 30대(15명), 40대(7명), 20대(6명) 순으로 많았으며, 20대 이상(30명) 성인 비율이 81.1%로 높게 나타났다. 큐열은 32명 발생하여 전년(17명) 대비 88.2% 증가했다. 특히 경기지역에서 환자가 많이 증가하여, 수도권역 발생 중 81.3%가 경기에서 발생했다. 전년 대비 가장 감소폭이 큰 감염병은 백일해로 2025년 2,133명 발생했고, 전년(26,247명) 대비 91.9% 감소했다. 백일해는 2024년 국내 최대 규모의 유행을 보였고 2025년 감소한 것으로 나타났다. 시도별로는 경기, 서울, 인천, 강원 순으로 많은 환자가 발생했다.

## 논 의

2025년 수도권역 전수감시 법정 감염병 신고환자 수는 총 74,895명(AIDS 제외)으로 전국 138,709명의 54.0%를 차지했다. 제1급 감염병(18종) 의심신고는 총 508건으로 이 중 의사환자는 72명이었고 확진 사례는 없었다. 제2급 감염병(21종)은 총 69,118명, 제3급 감염병(27종, AIDS 제외)은 총 5,777명 신고되었다.

수도권센터는 감염병별 위험도 및 특성 등을 고려하여 고위험군 및 집단발생의 중점사례와 주요 감염병의 집단사례를 관리하고 있다. 홍역은 (의사)환자 발생 신고사례 중 수도권역 환자는 37명으로 전체 환자(78명)의 47.4%를 수도권센터에서 대응하였고, 감염경로는 모두 해외유입 또는 해외유입 관련 사례였다. 세계보건기구(World Health Organization, WHO)에 의하면, 2025년 말 기준(전 세계 홍역 예방접종률), 생후 2세까지 홍역 1차 접종률(first dose of measles-containing vaccine, MCV1)은 84%, 2차 접종률(MCV2)은 77%로 홍역 전파 차단에 필요한 2회 접종 95% 수준에 크게 못 미치는 상황이다[2]. 반면, 우리나라의 2세 MMR (홍역, 유행성이하선염, 풍진) 접종률은 96.7%, 6세 MMR 접종률은 95.6%로 높게 나타났다[3]. 코로나19 이후 국제적 교류 및 해외여행 증가 등으로 인해 전 세계적으로 홍역 발생이 증가하는 상황에서 해외유입 사례가 지속 발생하여 신고건 전수감시 및 역학조사 기술 지원 등 조기 발견과 확산 차단을 위한 대응체계가 작동 중이다. 결핵 중점사례는 영유아 결핵 노출, 추가 환자 발생, 다제내성 도말양성 등으로 총 114건을 중점 관리하였다. CRE 감염증은 병원체보유자를 포함하여 수도권역 환자 31,113명으로, 전체 환자(49,053명)의 63.4%를 수도권센터에서 대응하였다. 말라리아 군집사례(2명 이상)는 총 25건으로 환자 발생 감시와 군집사례 전수에 대하여 센터-지자체 합동으로 대응하고 있다. 레지오넬라증은 인구 고령화, 건물 노후화, 기후 변화 등의 영향으로 신고건수가 증가하는 추세로, 2025년 수도권역 신고 환자수는 총 396명으로 전년(276명) 대비 43.5% 증가하였고, 전국(640명)의 61.9%를 차지하였다. 수도권센터는 특히 의료기관 내 환자 발생사례에 대하여 민관합동 역학조사, 전문가 자문회의, 중재조치와 환경감시 등 사례별로 대응 관리하고 있다.

수도권역에서 발생하는 감염병의 추세를 분석한 결과 다음과 같은 특성이 나타났다.

첫째, 의료관련감염병인 CRE 감염증의 지속적인 증가이다. 수도권역 내 상급종합병원은 25개소(전국 47개소), 요양병원은 700개소(전국 1,306개소)로 의료기관 수가 전국의 50% 이상을 차지하며 최근 CRE 감염증은 2023년 23,847명, 2024년 26,623명, 2025년 31,113명이 발생하여 전년 대비 평균 17.7%의 증가율을 보였다. 이는 국내 CRE 감염증은 환자뿐 아니라 병원체보유자도 신고 대상으로, 최근 의료기관의 적극적인 선제 검사 시행으로 병원체보유자 신고건이 증가하고 있으며, 당분간 전국적으로 증가 추세가 이어질 것으로 예상된다[4]. 수도권센터는 관할지역 의료기관의 현장 점검과 교육강화 등 의료관련감염 예방관리를 위한 대응조치들을 시행하고 있다.

둘째, 성매개감염병인 매독의 발생이 집중되고 있다. 매독은 2024년부터 전수감시체계(제3급 법정 감염병)로 전환되었고 수도권역에서 1,630건(전국 2,790건) 신고되었다. 2025년 수도권역의 매독 신고 환자는 총 1,357명으로 전년 대비 다소 감소했으나 전국(2,211명)의 61.4%를 차지했으며, 특히 성접촉이 활발한 젊은 층들이 많은 서울‧경기의 발생 비율이 높았다. 또한, 법정 감염병 중 성매개감염병으로 분류되지는 않으나 주로 성접촉에 의한 감염으로 발생하는 엠폭스는 2022년 4명 모두 수도권역에서 발생, 2023년 전국적인 유행(전국 151명, 수도권역 122명)을 거쳐 2024년 산발적인 발생(전국 17명, 수도권역 16명)을 보였고, 2025년 9월 WHO에서 국제공중보건위기상황 해제 이후 12월 국내에서는 Clade 1b형과 2b형 통합 관리체계로 전환하여 대응하고 있다. 2025년 수도권역에서 25명이 신고되어 전국(27명)의 92.6%로 높은 비중을 차지했다. 2025년까지 국내에서 신고된 엠폭스 환자는 모두 Clade 2b형이었다. AIDS는 주요 감염경로가 성접촉이나 질병관리청에서 에이즈지원시스템(HASNet)을 통하여 전국 신고 현황을 관리하고 있으므로 본 연구에서는 제외하였다. 앞으로도 성매개감염병에 대한 인식개선과 의료 접근성 향상, 예방수칙 교육홍보 등이 더 강화될 필요가 있다.

셋째, 해외유입 사례 감염병의 수도권역 발생 비중이 높다(보충 표 1; available online). 감염병 포털 해외유입 통계[5]에 따르면, 수도권역의 해외유입 사례는 2025년 총 411명으로 전국(633명) 대비 64.9%를 차지한다. 수도권역에서 해외유입 사례가 많은 감염병은 매독(138명), 뎅기열(69명), 홍역(32명) 순이었으며, 국내 발생 모두 해외유입인 뎅기열은 전국 110명 중 수도권역에서 69명 발생하여 62.7% 차지, 유비저는 전국의 해외유입 사례 5명 중 4명이 수도권역에서 발생하여 80.0%의 높은 비중을 보였다, 지카바이러스감염증은 3명(100%) 모두 수도권역 신고 사례였다. 해외유입 감염병의 선제적 예방을 위해 출국자 대상 캠페인 등에 대한 시민 접근성을 더 개선할 필요가 있다.

마지막으로 말라리아는 매년 전국 발생의 약 90%가 수도권역에서 발생하고 있다. 전국 대비 수도권역의 발생 비율은 2023년 91.4% (683/747명), 2024년 91.7% (654/713명), 2025년 89.5% (538/601명)로 수도권역에서 대부분 발생하고 있다. 국내에서 발생하는 삼일열말라리아는 경기북부, 인천 일부 지역에서 주로 발생하기 때문이다[4]. 라임병은 2025년 수도권역에서 29명 발생하여 전국(41명) 대비 70.7% 비중이며, SFTS는 전국(280명) 중 수도권역 90명으로 32.1%의 낮은 비중을 보였다. 이는 라임병과 SFTS 모두 참진드기 매개 감염병이나 SFTS는 작은소피참진드기가 매개하며, 우리나라에서 작은소피참진드기가 참진드기의 78.46%를 차지하는 절대우점종이므로[6] 지역적 분포 차이의 영향이 있었을 것으로 생각된다.

본 연구는 「2025 감염병 신고 현황 연보」를 기초자료로 관할 시도별 감염병(제1–3급) 발생 신고건수의 기술적 통계를 중심으로 수도권역 내 감염병의 추세와 특성을 분석하였다. 지역사회의 감염병 발생 규모와 분포 및 추세를 파악하여 유행을 조기에 발견하고, 고위험 지역‧집단을 확인하며, 예방관리사업과 방역자원의 우선순위를 결정하는 근거를 제공하는 데 공중보건학적인 의미가 있다. 앞으로도 수도권센터는 수도권역의 감염병 예방관리와 신속 대응을 위해 관할 4개 시도와 협력체계를 강화하고 공동의 목표와 전략을 공유하며 현장 중심의 즉각적인 대응으로 감염병의 유입을 차단하고 추가 확산 방지에 기여할 것이다.

## Figures and Tables

**그림 1:** 2025년 전국 대비 수도권역 감염병별 신고 현황 의료관련감염병: 카바페넴내성장내세균목(CRE) 감염증, 반코마이신내성 황색포도알균(VRSA) 감염증. 예방접종대상감염병: 수두, 유행성이하선염, 백일해, 폐렴구균감염증, 홍역, b형헤모필루스 인플루엔자, 파상풍. 호흡기감염병: 성홍열, 수막구균감염증, 레지오넬라증. 바이러스간염: A형간염, E형간염, B형간염, C형간염. 성매개감염병: 매독. 매개체감염병: 말라리아, 쯔쯔가무시증, 중증열성혈소판감소증(SFTS), 뎅기열, 신증후군출혈열, 라임병, 발진열, 렙토스피라증, 일본뇌염, 치쿤구니야열, 유비저, 지카바이러스감염증. 수인성‧식품매개감염병: 장출혈성대장균(EHEC) 감염증, 세균성이질, 비브리오패혈증, 파라티푸스, 장티푸스. 인수공통감염병: 크로이츠펠트–야콥병(CJD), 큐열, 브루셀라증. 기타: 한센병, 엠폭스. CRE=carbapenem-resistant *Enterobacterales*; VRSA=vancomycin-resistant *Staphylococcus aureus*; SFTS=severe fever with thrombocytopenia syndrome; EHEC=enterohemorrhagic *Escherichia coli*; CJD=Creutzfeldt–Jakob disease.

**표 1:** 수도권역 제1급 법정 감염병 의심신고 및 의사환자 현황(2024–2025년)

감염병명	2025년				2024년		
	의심신고a)	의사환자b)	(비율)		의심신고a)	의사환자b)	(비율)
계	508	72	(14.2)		511	44	(8.6)
에볼라바이러스병	9	2	(22.2)		4	0	(0.0)
마버그열	19	4	(21.1)		12	0	(0.0)
라싸열	-	-	-		1	0	(0.0)
크리미안콩고출혈열	10	3	(30.0)		1	0	(0.0)
남아메리카출혈열	-	-	-		-	-	-
리프트밸리열	-	-	-		6	0	(0.0)
두창	1	0	(0.0)		-	-	-
페스트	2	0	(0.0)		80	1	(1.3)
탄저	-	-	-		-	-	-
보톨리눔독소증	-	-	-		-	-	-
야토병	1	1	(100.0)		-	-	-
중증급성호흡기증후군(SARS)	-	-	-		-	-	-
중동호흡기증후군(MERS)	350	42	(12.0)		366	38	(10.4)
디프테리아	2	2	(100.0)		-	-	-
동물인플루엔자 인체감염증	107	18	(16.8)		41	5	(12.2)
신종인플루엔자	-	-	-		-	-	-
신종감염병증후군	-	-	-		-	-	-
니파바이러스감염증c)	7	0	(0.0)		-	-	-

단위: 건, %. 확진환자 없음. SARS=severe acute respiratory syndrome; MERS=Middle East respiratory syndrome. ^a)^의심신고: 보건소, 의료기관, 1339, 검역소 등에서 인지한 의심사례 수. ^b)^의사환자: 임상증상 및 역학적 연관성을 고려하여 사례분류되어 진단검사한 사례 수. ^c)^2025년 9월 8일 제1급 감염병 추가.

**표 2:** 수도권역 제1급 법정 감염병 시도별 의심신고 및 의사환자 현황(2024–2025년)

구분		계	서울	인천	경기	강원
2025년	의심신고	508	186	52	245	25
	(인구 100만 명당)	(18.4)	(19.8)	(17.2)	(18.0)	(16.6)
	의사환자	72	20	6	33	13
	비율	(14.2)	(10.8)	(11.5)	(13.5)	(52.0)
2024년	의심신고	511	177	38	286	10
	(인구 100만 명당)	(18.4)	(18.9)	(12.6)	(21.0)	(6.6)
	의사환자	44	9	5	26	4
	비율	(8.6)	(5.1)	(13.2)	(9.1)	(40.0)

단위: 건, 인구 100만 명당 발생률, %.

**표 3:** 2025년 수도권역 법정 감염병 전수감시 신고 현황(시도별)

구분	질병명		신고수														
			전국	수도 권역	비중	서울			인천			경기			강원		2024년 수도권역
						신고수	발생률		신고수	발생률		신고수	발생률		신고수	발생률	
2급	결핵		17,070	8,216	48.1	2,853	30.9		928	30.7		3,732	27.3		703	46.6	8,763
	수두		30,248	14,904	49.3	3,592	38.56		1,293	42.58		8,966	65.39		1,053	69.59	14,567
	홍역		78	37	47.4	17	0.18		2	0.07		16	0.12		2	0.13	17
	장티푸스		33	8	24.2	1	0.01		1	0.03		6	0.04		-	0.00	20
	파라티푸스		24	12	50.0	7	0.08		-	0.00		3	0.02		2	0.13	9
	세균성이질		64	32	50.0	19	0.20		2	0.07		11	0.08		-	0.00	24
	장출혈성대장균 감염증		531	301	56.7	95	1.02		37	1.22		161	1.17		8	0.53	115
	A형간염		1,141	588	51.5	183	1.96		63	2.07		312	2.28		30	1.98	638
	백일해		5,491	2,133	38.8	486	5.22		256	8.43		1,195	8.71		196	12.95	26,247
	유행성이하선염		6,720	3,494	52.0	890	9.55		377	12.42		1,981	14.45		246	16.26	3,323
	수막구균감염증		12	6	50.0	1	0.01		-	0.00		5	0.04		-	0.00	15
	b형헤모필루스 인플루엔자		1	-	-	-	0.00		-	0.00		-	0.00		-	0.00	-
	폐렴구균감염증		440	266	60.5	106	1.14		27	0.89		120	0.88		13	0.86	276
	한센병		3	2	66.7	-	0.00		-	0.00		2	0.01		-	0.00	2
	성홍열		13,113	7,465	56.9	1,684	18.08		1,173	38.63		4,152	30.28		456	30.14	4,532
	반코마이신내성 황색포도알균(VRSA) 감염증		2	1	50.0	-	0.00		1	0.00		-	0.00		-	0.00	1
	카바페넴내성장내세균목(CRE) 감염증		49,053	31,113	63.4	12,553	134.75		3,887	128.01		13,464	98.19		1,209	79.90	26,623
	E형간염		915	540	59.0	183	1.96		37	1.22		277	2.02		43	2.84	449
3급	파상풍		24	8	33.3	1	0.01		2	0.07		5	0.04		-	0.00	17
	B형간염(급성)		226	103	45.6	33	0.35		11	0.36		53	0.39		6	0.40	125
	일본뇌염		7	5	71.4	1	0.01		-	0.00		3	0.02		1	0.07	14
	C형간염		5,639	2,457	43.6	790	8.48		472	15.54		1,076	7.85		119	7.86	2,865
	말라리아		601	538	89.5	70	0.75		108	3.56		332	2.42		28	1.85	654
	레지오넬라증		640	396	61.9	168	1.80		35	1.15		177	1.29		16	1.06	276
	비브리오패혈증		68	28	41.2	9	0.10		5	0.16		13	0.09		1	0.07	30
	발진열		16	9	56.3	1	0.01		5	0.16		3	0.02		-	0.00	28
	쯔쯔가무시증		3,405	510	15.0	105	1.13		56	1.84		330	2.41		19	1.26	701
	렙토스피라증		53	7	13.2	1	0.01		-	0.00		6	0.04		-	0.00	8
	브루셀라증		4	1	25.0	-	0.00		-	0.00		1	0.01		-	0.00	1
	신증후군출혈열		230	48	20.9	8	0.09		9	0.30		27	0.20		4	0.26	58
	크로이츠펠트–야콥병		99	53	53.5	22	-		7	-		22	-		2	-	43
	뎅기열		110	69	62.7	27	0.24		11	0.23		29	0.16		2	0.13	137
	큐열		72	32	44.4	3	0.29		1	0.36		26	0.21		2	0.13	17
	라임병		41	29	70.7	5	0.03		-	0.03		23	0.19		1	0.13	17
	유비저		5	4	80.0	2	0.05		-	0.00		1	0.17		1	0.07	1
	치쿤구니야열		9	5	55.6	4	0.02		1	0.00		-	0.01		-	0.07	7
	중증열성혈소판 감소증후군(SFTS)		280	90	32.1	14	0.04		3	0.03		42	0.00		31	0.00	74
	지카바이러스 감염증		3	3	100.0	1	0.15		-	0.10		2	0.31		-	2.05	-
	매독	1기	721	440	61.0	189	0.01		60	0.00		177	0.01		14	0.00	569
		2기	449	290	64.6	144	2.03		38	1.98		105	1.29		3	0.93	334
		3기	50	32	64.0	7	1.55		3	1.25		22	0.77		-	0.20	29
		선천성	12	8	66.7	5	0.08		2	0.10		1	0.16		-	0.00	3
		(조기) 잠복	979	587	60.0	236	0.05		85	0.07		248	0.01		18	0.00	695
	엠폭스		27	25	92.6	14	2.53		2	2.80		9	1.81		-	1.19	16

단위: 명, %, 인구 10만 명당 발생률. 각 질병별로 규정된 신고범위(환자, 의사환자, 병원체보유자)의 모든 신고건 포함. 환자 주소지 기준분류로 실제 추정감염지역과 다를 수 있음(단, VRSA 감염증, CRE 감염증은 의료기관 주소지 기준, 말라리아는 군인환자의 경우 군부대 주소지 보건소[관리보건소] 기준임). 2025년 환자 신고가 있는 감염병에 한해 작성함. 후천성면역결핍증은 개인정보보호 및 지역에 대한 편견으로 지역별 통계 미제공. VRSA=vancomycin-resistant *Staphylococcus aureus*; CRE=carbapenem-resistant *Enterobacterales*; SFTS=severe fever with thrombocytopenia syndrome.
